# Supplementary material for: Planktonic foraminifera regulate calcification according to ocean density
Source: Commun Earth Environ. 2025 Jul 31;6(1):605. doi: 10.1038/s43247-025-02558-w (PMC12313521; doi:10.1038/s43247-025-02558-w)
Supplement: Supplementary file 2 — Supplementary Information [file 43247_2025_2558_MOESM2_ESM.pdf]

Supplementary Information for

Planktonic foraminifera regulate calcification according to ocean density

Stergios D. Zarkogiannis, James W. B. Rae, Benjamin R. Shipley, P. Graham Mortyn

Corresponding author: Stergios Zarkogiannis

Email: stergios.zarkogiannis@earth.ox.ac.uk

This PDF file includes:

Supplementary Figures 1 to 3

Supplementary Tables 1 to 5

Supplementary Note

Supplementary References

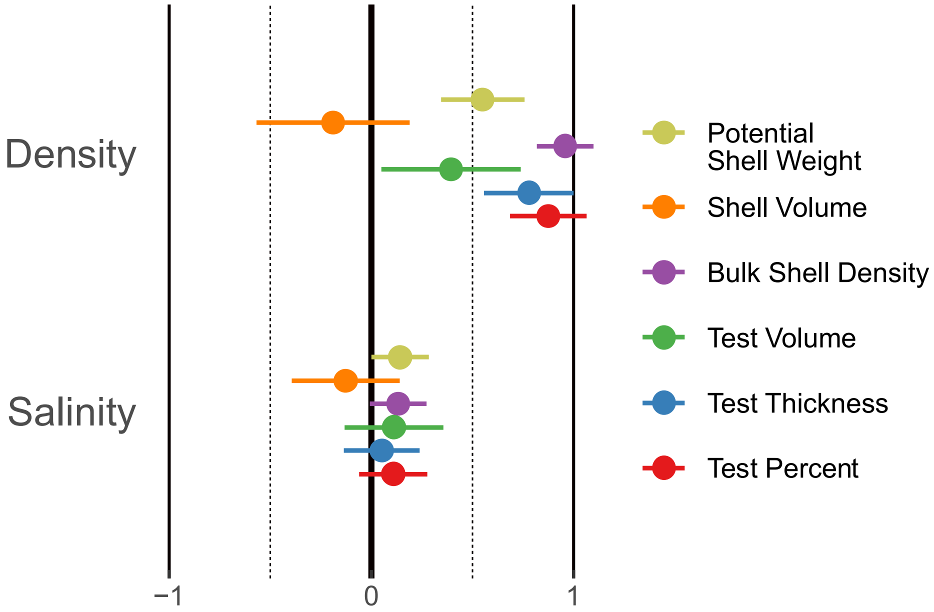

Supplementary Figure 1. Coefficients (dots) and standard errors (lines) for the linear mixed effect models examining the relationship between each morphological trait and density and salinity. Species identity was held as a random effect, and all fixed effects and responses were centered and scaled to unit variance. Insignificant effects cross the black vertical line at 0.

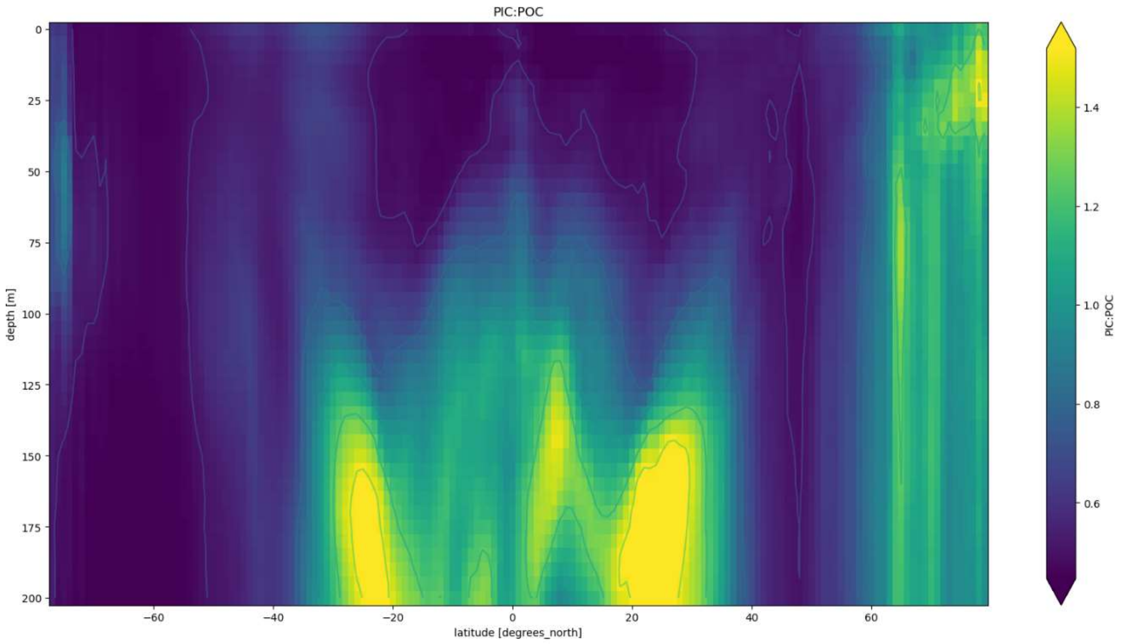

Supplementary Figure 2. Total coccolithophore PIC:POC ratios derived from integrated model output. The data represent predictions from 85 species-specific species distribution models (SDMs) mapped onto a global Latitude/Longitude/Depth/Month grid from CASCADE dataset[1].

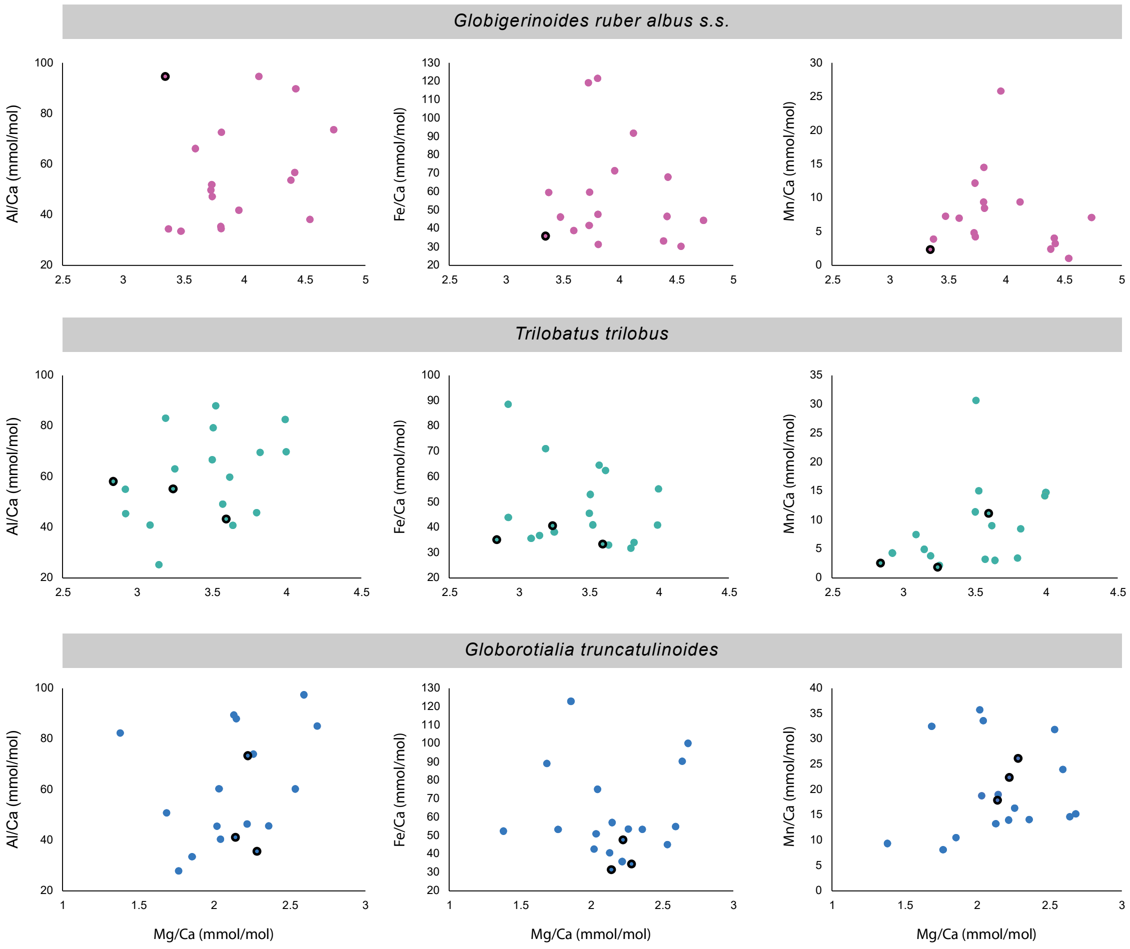

Supplementary Figure 3. Al/Ca (left), Fe/Ca (middle) and Mn/Ca (right) of each species and sample versus Mg/Ca. Note the different scaling of the axes for individual species. Results from replicate measurements are shown separately.

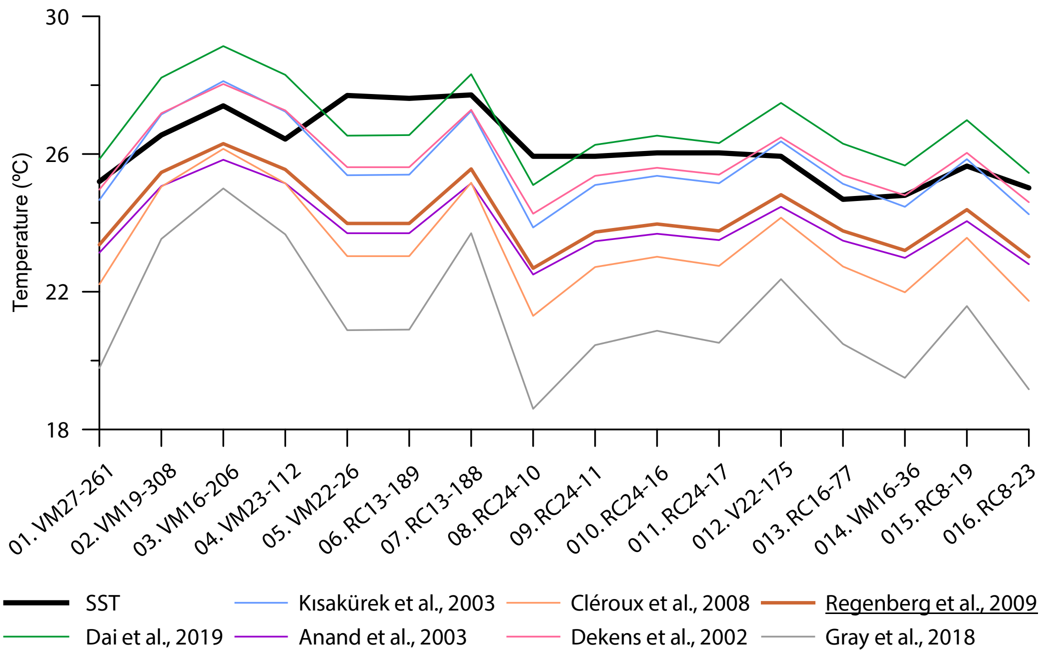

Supplementary Figure 4. Comparison of different sea surface temperature (SST) estimates derived from published Mg/Ca-temperature equations with Argo in situ SST measurements.

Supplementary Table 1. Spearman's rank correlation coefficients for each of the traits used in the study.

	Shell Weight	"Potential" cell volume	Bulk shell density	Test Volume	Test Thickness	Test Percent
Shell Weight	1	0.452	0.839	0.852	0.878	0.871
"Potential" cell volume	0.452	1	-0.028	0.729	0.318	0.275
Bulk shell density	0.839	-0.028	1	0.532	0.786	0.819
Test Volume	0.852	0.729	0.532	1	0.847	0.830
Test Thickness	0.878	0.318	0.786	0.847	1	0.977
Test Percent	0.871	0.275	0.819	0.830	0.977	1

Supplementary Table 2: AICc values for each linear model between species traits and environmental predictors, treating each species individually: a) *G. ruber*, b) *T. trilobus*, c) *G. truncatulinoides*. The best-performing model (lowest AICc) is bolded, along with all other models with ΔAIC < 2. Full = Density + Salinity + Temperature + $[CO_3^{2-}]$. All variables were centered and scaled before running the models and calculating the AICc values.

**a) *Globigerinoides ruber albus* s.s.**						
Model	Potential Shell Volume	Shell Weight	Bulk Shell Density	Test Volume	Test Thickness	Test Percent
Density	**43.73**	**27.72**	**31.73**	47.36	47.94	41.59
Salinity	45.78	34.51	38.57	**39.76**	35.34	**34.44**
Temperature	48.41	48.05	47.61	46.85	49.19	48.59
Density + Salinity	47.39	**27.94**	34.27	42.38	**32.67**	37.97
Density + $[CO_3^{2-}]$	47.12	**28.79**	35.48	42.51	41.57	38.85
Salinity + $[CO_3^{2-}]$	46.00	37.55	39.39	40.82	36.56	37.83
[CO3]	49.66	45.81	48.15	**39.41**	38.04	41.68
Temp +$[CO_3^{2-}]$	52.18	47.67	49.76	**37.77**	41.01	43.87
Full	54.54	36.38	41.32	41.96	37.44	45.81
**b) *Trilobatus trilobus***						
Model	Potential Shell Volume	Shell Weight	Bulk Shell Density	Test Volume	Test Thickness	Test Percent
Density	52.34	40.95	**45.09**	47.1	41.04	39.02
Salinity	51.72	**33.74**	**44.63**	39.03	**25.6**	**24.93**
Temperature	**48.66**	51.82	52.2	49.69	51.91	51.86
Density + Salinity	55.07	**35.28**	**46.63**	42.66	27.86	**25.55**
Density + $[CO_3^{2-}]$	51.43	38.09	48.58	36.61	**26.78**	31.74
Salinity + $[CO_3^{2-}]$	50.9	37.24	46.89	36.73	**25.08**	28.09
Temp + $[CO_3^{2-}]$	**48.18**	44.22	50.99	36.35	36.61	41.57
$[CO_3^{2-}]$	**47.25**	47.16	54.39	**32.9**	39.41	44.47
Full	55.27	43.09	54.87	35.23	28.01	31.05
c) Globorotalia truncatulinoides using $ACD_{Mg/Ca}$						
Model	Potential Shell Volume	Shell Weight	Bulk Shell Density	Test Volume	Test Thickness	Test Percent
Density	50.08	**44.98**	**42.71**	52.01	**47.91**	**46.74**
Salinity	**45.3**	52.26	48.73	**49.03**	51.73	52.24
Temperature	48.66	51.37	48.77	51.99	52.11	51.86
Density + Salinity	47.5	48.54	**43.35**	51.33	**49.22**	49.22
Density + $[CO_3^{2-}]$	50.7	48.36	**43.6**	53.14	50.02	49.23
Salinity + $[CO_3^{2}]$	48.65	54.78	52.3	52.62	55.36	55.79
Temp + $[CO_3^{2}]$	48.8	51.64	49.38	**50.37**	51.93	52.16
$[CO_3^{2}]$	52.1	55	52.37	52.17	**48.92**	48.95
Full	56.84	53.79	50.82	59.93	56.34	56.49

d) Globorotalia truncatulinoides using ACD_{iso}						
Model	Potential Shell Volume	Shell Weight	Bulk Shell Density	Test Volume	Test Thickness	Test Percent
Density	50.08	**44.83**	42.71	52.01	**47.91**	**46.74**
Salinity	50.61	51.07	52.37	**47.88**	**47.84**	49.23
Temp	51.71	51.2	52.26	50.02	**49.72**	50.02
Density + Salinity	**46.84**	48.47	**42.04**	51.36	**49.44**	49.48
Density + [CO_3^{2-}]	**48.46**	47.65	**40.35**	52.01	**49.65**	49.18
Salinity + [CO_3^{2-}]	54.21	54.08	55.97	51.4	51.11	52.14
Temp + [CO_3^{2}]	51.3	52.04	52.35	**48.52**	**47.92**	48.56
[CO_3^{2}]	54.65	52.48	53.51	51.08	50.13	51.16
Full	56.37	53.25	48.64	59.88	55.87	55.9

Supplementary Table 3. List of R Packages used, their version number, and the citation.

Package	Version	Citation
lme4	1.1	Bates et al.[2]
MuMin	1.47	Bartoń[3]
car	3.1	Fox and Weisberg[4]
sjPlot	2.8	Lüdecke[5]
lmtest	0.9	Zeileis and Hothorn[6]

Supplementary Note

In species such as *G. truncatulinoides*, and as is typical across crust-forming taxa, the dense gametogenic crust calcite tends to exhibit lower Mg/Ca ratios than the earlier, ontogenetic calcite, regardless of actual calcification depth[7-9]. Consequently, using Mg/Ca ratios alone to estimate calcification depth may introduce bias, as more heavily calcified individuals bearing more extensive crust may appear to have calcified deeper in the water column than they truly did.

To evaluate whether this potential bias affects our results, we conducted additional statistical analyses using oceanographic parameters extracted at calcification depths estimated from $\delta^{18}O_{G.truncatulinoides}$, rather than Mg/Ca. Specifically, Argo temperature profiles were converted into $\delta^{18}O_{sw}$ values using the quadratic paleotemperature equation of Kim and O'Neil[10], as reformulated by Bemis et al.[11]. Isotope-based apparent calcification depths (ACD_{iso}) were then estimated by matching the measured $\delta^{18}O$ of *G. truncatulinoides* shells to the seawater profile, under the assumption of calcification in isotopic equilibrium with ambient seawater for this species[12-14] and can thus reflect accurate calcification depths. The *G. truncatulinoides* $ACDs_{iso}$ along with the oceanographic parameters extracted at these depths are given in Supplementary Table 4.

We then re-ran both the individual species models and linear mixed model (LMM) using these isotope-based depths for *G. truncatulinoides*, while retaining $ACD_{Mg/Ca}$ for the other two species (see Supplement Table 2d and Supplementary Table 5 accordingly). When using ACD_{iso}, the best-performing models remained largely consistent with the original analysis: density-based predictors continued to perform best for most traits. The only exception was for test volume, where the model including temperature and $[CO_3^{2-}]$ slightly outperformed density-based models (Supplementary Table 3). For the individual species models, density + salinity and density + $[CO_3^{2-}]$ remained the best predictors for potential cell volume and BSD, while temperature-based models showed better performance for test volume and test thickness (Supplement Table 2d). Importantly, the AIC rankings and overall model selection changed negligibly between Mg/Ca and $\delta^{18}O$-based depth estimates (Supplementary Table 5).

These findings confirm that the use of Mg/Ca-based depth estimates, despite potential biases in crust-forming species like *G. truncatulinoides*, does not alter the central conclusions of the study. Moreover, the Mg/Ca-based estimates used here rely on a region- and species-specific calibration by Cléroux et al.[15], which was derived from bulk (crust + ontogenetic) shells collected in the same Atlantic region. This calibration inherently accounts for the low-Mg crustal contribution and reflects an integrated signal across the full shell. Following the recommendation of Hupp and Fehrenbacher[8] that crust-forming taxa be treated with calibrations developed from crusted tests, this approach offers a robust estimate of calcification depth for crust-forming taxa and minimizes potential depth misassignments related to shell layering.

Both Mg/Ca and $\delta^{18}O$ methods carry uncertainties and potential systematic biases. While such biases may affect absolute depth estimates, comparisons remain valid when all observations are derived using the same method. In our statistical models, however, using different approaches to estimate depth introduces unquantifiable differences that could confound comparisons between species. Therefore, to ensure methodological consistency across all species, maintain internal comparability and minimize uncertainties only the Mg/Ca-derived depth estimates are reported in the main text.

Supplementary Table 4: Summary table listing all the *G. truncatulinoides* shell traits calculated, together with the associated geochemical and *in situ* oceanographic data at ACD_{iso}. Temperatures were converted from the Mg/Ca data via the calibration equation of ref[16]. Daggers (†) denote a distinct G. truncatulinoides group. BSD = Bulk shell density and ACD = calcification depth. Refer to the Methods for a complete description of the parameters.

Sample Location	Species	Shell weight (µg)	Cell Volume (µm³)	Test Volume (µm³)	Test %	Test thickness (µm)	BSD (g/cm³)	$\delta^{18}O_{shell}$ (‰)	Mg/Ca (mmol/mol)	$Temperature_{Mg/Ca}$ (°C)	$Depth_{Mg/Ca}$ (m)	Salinity at ACD_{iso}	CO_3^{2-} (µmol/kg)	Alkalinity (µmol/kg)	$Density_{Mg/Ca}$ (kg/m³)	3D Salinity	3D Density	3D Temp. (°C)
1. VM27-261	*G. truncatulinoides*	28.5	17,060,150	6,974,236	41%	7.5	1.67	2.59	2.594 (2.224)	16.3	363	36.2	199.7	2,356	1027.6	36.0	26.9	14.6
2. VM19-308	*G. truncatulinoides*†	29.9	17,449,790	7,854,978	45%	8.5	1.72	2.22	2.218 (2.142)	13.7	305	36.4	216.2	2,386	1027.0	36.4	26.6	17.1
3. VM16-206	*G. truncatulinoides*†	32.1	17,778,270	8,943,279	49%	9.9	1.80	2.02	2.020	12.1	543	35.8	158.4	2,345	1027.7	35.8	27.0	13.1
4. VM23-112	*G. truncatulinoides*	33.2	16,765,850	8,201,975	48%	9.4	1.98	2.54	2.536	15.9	449	35.6	135.7	2,320	1028.4	35.2	27.2	10.2
5. VM22-26	*G. truncatulinoides*†	32.6	19,037,260	9,569,991	50%	10.1	1.71	1.69	1.688	9.1	277	34.9	108.0	2,306	1027.7	35.0	27.0	10.0
6. RC13-189	*G. truncatulinoides*	32.0	18,121,580	9,407,324	52%	10.3	1.77	2.26	2.261	14.0	312	35.0	109.1	2,310	1028.2	35.0	26.9	10.4
7. RC13-188	*G. truncatulinoides*†	31.0	18,230,720	8,706,790	47%	9.3	1.70	2.13	2.130	13.0	167	35.4	159.4	2,331	1027.4	35.3	26.6	13.6
8. RC24-10	*G. truncatulinoides*†	27.6	19,862,900	9,237,238	47%	9.2	1.39	1.38	1.382	5.8	339	35.0	97.6	2,314	1026.8	34.9	27.0	9.5
9. RC24-11	*G. truncatulinoides*	31.3	16,298,344	8,095,077	49%	9.4	1.92	1.86	1.856	10.7	438	34.7	84.4	2,307	1028.4	34.7	27.2	8.1
10. RC24-16	*G. truncatulinoides*	32.0	17,094,350	8,708,787	51%	10.1	1.87	2.36	2.360	14.7	316	34.9	92.1	2,307	1028.6	34.9	27.0	9.9
11. RC24-17	*G. truncatulinoides*	29.7	19,931,571	8,957,021	46%	9.4	1.49	1.77	1.767	9.9	342	34.8	87.7	2,305	1028.0	34.9	27.0	9.4
12. V22-175	*G. truncatulinoides*	31.1	17,762,000	9,219,448	52%	10.4	1.75	2.68	2.682	16.9	176	35.3	177.0	2,332	1028.0	35.3	26.6	15.1
13. RC16-77	*G. truncatulinoides*	32.0	17,269,650	8,008,652	46%	9.2	1.85	2.03	2.034	12.3	314	34.9	112.7	2,299	1028.2	34.9	26.9	9.9
14. VM16-36	*G. truncatulinoides*	30.6	17,853,390	8,339,591	46%	9.1	1.72	2.64	2.640	16.6	317	35.0	132.9	2,311	1028.1	35.0	26.8	11.6
15. RC8-19	*G. truncatulinoides*†	30.1	17,283,360	7,202,748	41%	7.7	1.74	2.04	2.044 (2.283)	12.3	296	35.3	170.2	2,329	1027.2	35.2	26.5	13.7
16. RC8-23	G. truncatulinoides†	35.0	19,380,470	9,276,164	47%	9.6	1.81	2.15	2.147	13.2	435	34.9	149.8	2,304	1028.0	34.9	26.8	10.9

Supplementary Table 5: Akaike information criterion (AICc) values for each linear mixed-effects model (LMM), with species as a random intercept when using ACD_{iso} for *G. truncatulinoides*. The best-performing model (lowest AICc) is bolded, along with all other models with ΔAICc < 2. Full = density + salinity + Temperature + [CO_3^{2-}]. All variables were centered and scaled before running the models and calculating the AICc values. AICc values for the linear models using each species individually can be found in Supplementary Table 2d.

Model	Potential Cell Volume	Shell Weight	Bulk Shell Density	Test Volume	Test Thickness	Test Percent
Density	**96.2**	**39.05**	**66.36**	90.86	**82.04**	**73.34**
Salinity	**97.8**	64.26	102.53	95.06	96.24	97.75
Density + Salinity	98.41	**38.37**	**66.14**	92.19	84.44	**73.8**
Density + [CO_3^{2-}]	98.69	**38.67**	**66.83**	92.95	84.49	**75.23**
Salinity + [CO_3^{2-}]	98.91	61.96	97.21	94.96	90.85	88.83
Temp	98.51	72.64	105.4	93.31	94.89	99.73
[CO_3^{2-}]	99.08	75.29	111.76	99.27	102.63	107.69
Temp + [CO_3^{2-}]	100.54	64.58	104.55	**85.11**	92.24	96.03
Full	99.42	**38.69**	71.41	**85.61**	**82.76**	**73.69**

Supplementary References

1. de Vries J*, et al.* CASCADE: Dataset of extant coccolithophore size, carbon content and global distribution. *Scientific Data* **11**, 920 (2024).

2. Bates D, Mächler M, Bolker B, Walker S. Fitting Linear Mixed-Effects Models Using lme4. *Journal of Statistical Software* **67**, 1 - 48 (2015).

3. Bartoń K. MuMIn: Multi-model inference [Computer software]. R package version 1.43. 17. (2020).

4. Fox J, Weisberg S. Visualizing Fit and Lack of Fit in Complex Regression Models with Predictor Effect Plots and Partial Residuals. *Journal of Statistical Software* **87**, 1 - 27 (2018).

5. Lüdecke D. sjPlot: data visualization for statistics in social science.–R package ver. 2.8. 14. (2023).

6. Zeileis A, Hothorn T. Diagnostic Checking in Regression Relationships. *R News* **2**, 7-10 (2002).

7. Reynolds CE, Richey JN, Fehrenbacher JS, Rosenheim BE, Spero HJ. Environmental controls on the geochemistry of Globorotalia truncatulinoides in the Gulf of Mexico: Implications for paleoceanographic reconstructions. *Mar Micropaleontol* **142**, 92-104 (2018).

8. Hupp BN, Fehrenbacher JS. Geochemical Differences Between Alive, Uncrusted and Dead, Crusted Shells of *Neogloboquadrina pachyderma*: Implications for Paleoreconstruction. *Paleoceanography and Paleoclimatology* **38**, e2023PA004638 (2023).

9. Davis CV, Fehrenbacher JS, Hill TM, Russell AD, Spero HJ. Relationships Between Temperature, pH, and Crusting on Mg/Ca Ratios in Laboratory-Grown *Neogloboquadrina* Foraminifera. *Paleoceanography* **32**, 1137-1152 (2017).

10. Kim S-T, O'Neil JR. Equilibrium and non-equilibrium oxygen isotope effects in synthetic carbonates. *Geochim Cosmochim Acta* **61**, 3461-3475 (1997).

11. Bemis BE, Spero HJ, Bijma J, Lea DW. Reevaluation of the oxygen isotopic composition of planktonic Foraminifera: Experimental results and revised paleotemperature equations. *Paleoceanography* **13**, 150-160 (1998).

12. Erez J, Honjo S. Comparison of isotopic composition of planktonic foraminifera in plankton tows, sediment traps and sediments. *Palaeogeogr, Palaeoclimatol, Palaeoecol* **33**, 129-156 (1981).

13. Lončarić N, Peeters FJC, Kroon D, Brummer G-JA. Oxygen isotope ecology of recent planktic foraminifera at the central Walvis Ridge (SE Atlantic). *Paleoceanography* **21**, n/a-n/a (2006).

14. Rebotim A, Voelker AHL, Jonkers L, Waniek JJ, Schulz M, Kucera M. Calcification depth of deep-dwelling planktonic foraminifera from the eastern North Atlantic constrained by stable oxygen isotope ratios of shells from stratified plankton tows. *Journal of Micropalaeontology* **38**, 113-131 (2019).

15. Cléroux C, deMenocal P, Arbuszewski J, Linsley B. Reconstructing the upper water column thermal structure in the Atlantic Ocean. *Paleoceanography* **28**, 503-516 (2013).

16. Regenberg M, Steph S, Nürnberg D, Tiedemann R, Garbe-Schönberg D. Calibrating Mg/Ca ratios of multiple planktonic foraminiferal species with δ^{18}O-calcification temperatures: Paleothermometry for the upper water column. *Earth Planet Sci Lett* **278**, 324-336 (2009).
